# Comparative Analysis of the Wounded in Patients and Deaths in a Hospital Following the Three Major Earthquakes in Western China

**DOI:** 10.3389/fpubh.2022.775130

**Published:** 2022-07-08

**Authors:** Shan Xu, Bo Shi, Jianbo Yuxian, Mei He, Pei Yang, Weiyun Xu, Gang Liu, Zhongjin Song, Xiaobo Du, Dong Wang

**Affiliations:** ^1^Department of Disaster Medicine Research Group, MianYang Central Hospital, Mianyang, China; ^2^Department of Orthopedics, MianYang Central Hospital, Mianyang, China; ^3^Deparment of General Surgery, MianYang Central Hospital, Mianyang, China; ^4^Department of Nursing, MianYang Central Hospital, Mianyang, China; ^5^Department of Hepatobiliary Surgery, MianYang Central Hospital, Mianyang, China; ^6^Department of Breast Surgery, MianYang Central Hospital, Mianyang, China; ^7^Department of Science and Education, MianYang Central Hospital, Mianyang, China

**Keywords:** earthquake, disaster medicine, medical rescue, first aid, disaster management

## Abstract

The purpose of this study was to analyze the injury characteristics of patients and therapeutic strategies for patients injured in the last three big earthquakes in China, so as to provide a reference for the improvement of emergency plans for earthquakes. The analysis was based on the data provided by the Mianyang Central Hospital (MCH) from May 12th, 2008 to September 26th, 2017. Microsoft EXCEL software was used for data input, and SPSS was used for statistical analysis. A total of 1,390 earthquake-related patients were hospitalized in MCH. Most patients were admitted to the hospital within the first 2 weeks after the earthquake. The main causes for seismic injuries involved hit/strike by objects or building collapse /burying. Extremity fractures accounted for most injuries, especially 3 days after an earthquake. But soft tissue injuries cannot be neglected. Most earthquake patients were mainly treated by means of surgery and the majority were related to orthopedics. We found that different areas, population, and religions needed a tailored approach to the rescue effort. Therefore, the earthquake magnitude scale has a significant influence on mechanisms, types and severity of the injury of patients injured in earthquakes, as well as their timely transfer, management, and prognosis. Traumatic injuries are very common and thereby various surgical procedures especially orthopedic and neurosurgery are the domain of treatment modalities. Disaster preparedness and combined surgical team effort need to be focused on to reduce both mortality and morbidity.

## Introduction

**Figure 1:**
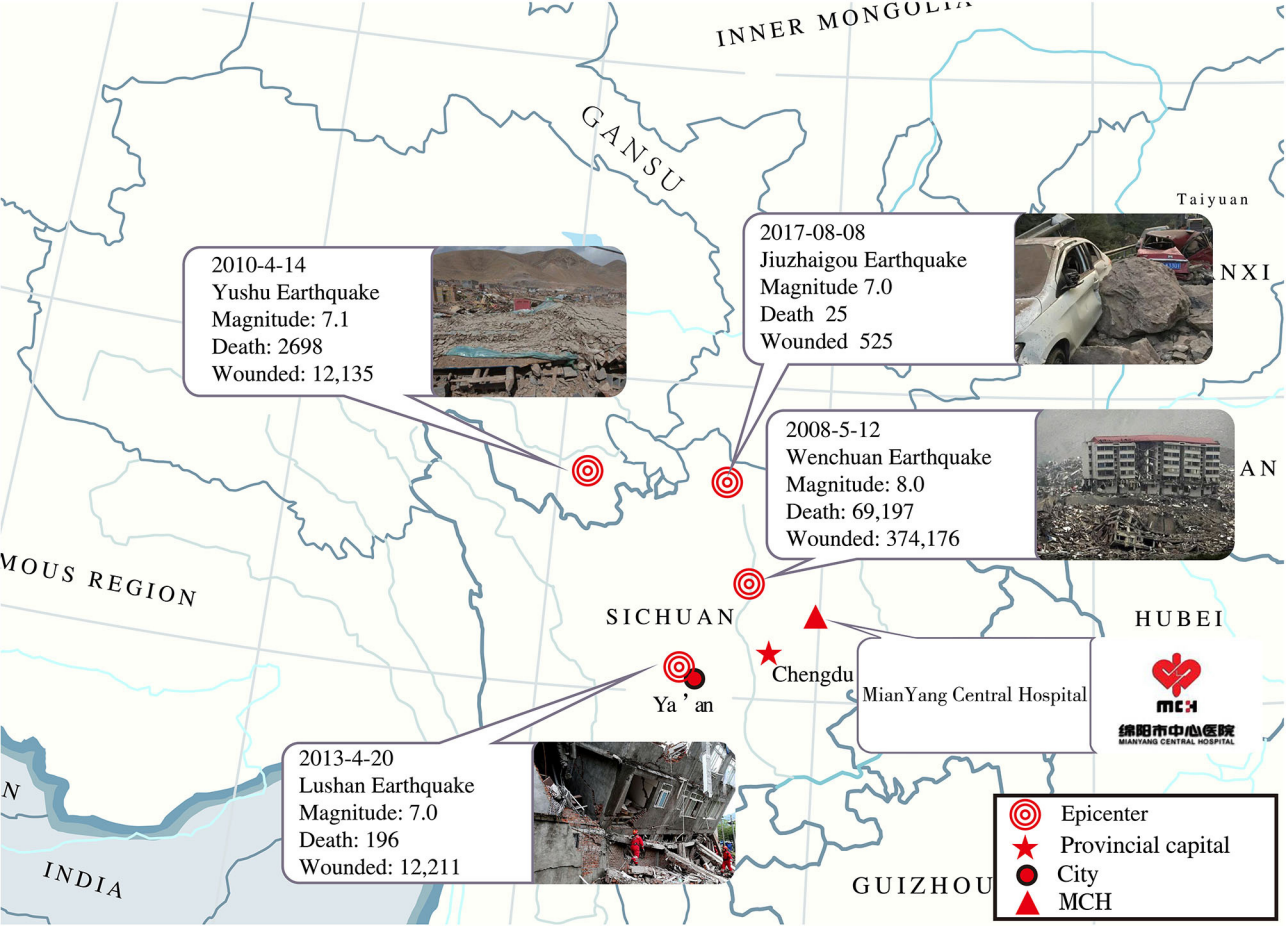
Overview of four catastrophic earthquakes in the past 10 years in China.

**Figure 2:**
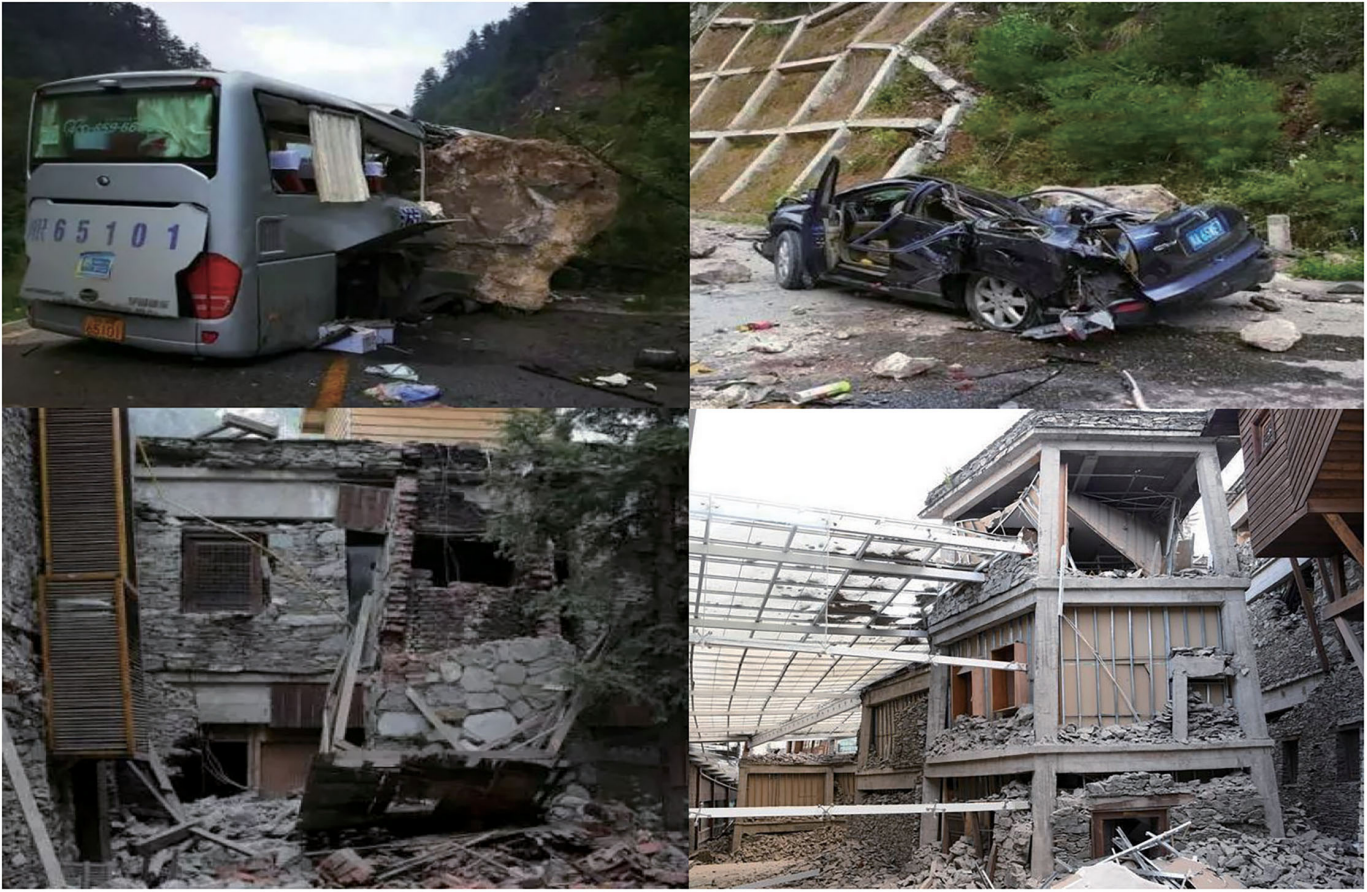
Scenes in Jiuzhaigou after the earthquake.

On August 8th, 2017, at 9:19 pm China Standard Time, a Ms 7.0 earthquake struck the Aba prefecture government in the Sichuan province of China. The seismic intensity was higher than level IX on the Mercalli intensity scale, and the earthquake epicenter in Zhangzha County could reach level VIII (1). In Western China, this earthquake was the fourth strongest earthquake in the past decade, such as Ms 8.0 Wenchuan Earthquake at 2:28 pm China Standard Time on May 12th 2008. A maximum shaking intensity of XI on the Mercalli intensity scale for this area (2). Ms 7.0 Yushu earthquake at 7:49 am on April 14th in 2010 (3). It had a maximum shaking intensity of IX on the Mercalli intensity scale. The next one is Ms 7.0 Yaan earthquake on April 20th in 2013 (4) (Figure 1). The epicenter was about 39 kilometers (24 miles) from the city of Jiuzhaigou. Jiuzhaigou is a famous scenic spot in China (5), which is visited by about 3.6 million tourists annually (6). The Jiuzhaigou earthquake caused major landslides, building collapses, traffic destruction, communication failure, and destructed numerous medical units (Figure 2), which significantly increased the difficulty of the medical rescue. Moreover, the Jiuzhaigou earthquake occurred in a tourist area and lacked adequate medical equipment and facilities. This disaster has claimed the lives of over 29 individuals suffering from 543 injuries. Over 500 patients were evaluated and treated in the hospitals available and most of the earthquake victims were tourists.

The Mianyang Central Hospital (MCH), a national-level academic medical center in Sichuan Province, China, is one of the largest city hospitals in the Sichuan province with 1,500 sickbeds. After the Jiuzhaigou earthquake, The MCH immediately launched an emergency response the earthquake sent a rescue team including doctors, nurses, and security experts to the area that was hit by the earthquake, and established field hospitals in 2 days. From August 9th, 2017 to August 11th, 2017, a total of 48 earthquake victims were transferred to the MCH or field hospitals, and all were hospitalized. So far, the MCH had taken part in the rescue after 3 earthquakes and admitted roughly 2,000 earthquake patients from Wenchuan, Yushu, and Jiuzhaigou earthquakes.

Although earthquakes are natural disasters that cannot be prevented or forecasted, the damage can be minimized with effective disaster planning as well as successful emergency rescues. Therefore, comprehensive research on the cause, characteristics, and treatment of injuries caused by earthquakes is needed. For example, a lot of victims in earthquakes suffer injuries that need surgical intervention, but the main gaps in knowledge about the earthquake epidemiology of earthquakes compromise the surgical delivery. What is the distribution and burden of surgical conditions following earthquakes? According to the studies after the 1988 Armenian earthquake, the head, superficial and lower extremity injury occupied most of the cases (7). Furthermore, other researchers highlighted the significance of fracture and crush injury, which usually represents over half of the injuries recorded (8, 9). Thus, more information concerning the profiles and categories of these injuries is required, which can help better deal with surgical management of the wounded after an earthquake.

In our study, we retrospectively reviewed the clinical parameters and treatment measures of earthquake patients after three large earthquakes occurred in different geolocations, such as mountain valleys and cities, with different crowds, including tourists and residents, and different ground shaking intensities. In conclusion, our study will draw lessons for the future, and determine effective measures to better deal with different earthquake victims under different circumstances.

## Methods

### Subjects and Study Design

This is a retrospective study of the clinical characteristics of 48 Jiuzhaigou earthquake patients that were collected between August 9th, 2017 and August 11th, 2017, 160 Yushu earthquake patients between April 16th, 2010 and April 20th, 2010, and 1,182 Wenchuan earthquake patients between May 12th 2008 and June 30th 2008. In this retrospective study, a total of 1,390 earthquake-related patients in the MCH and MCH field hospitals were reviewed. Most patients were sent to the hospital within the first 2 weeks after the earthquake. Inclusion criteria were as follows: patients who were directly or indirectly injured by Jiuzhaigou, Yushu, or Wenchuan earthquakes. Patients with non-injury-related diseases, such as stress disorder, delivery, and internal diseases, or not hospitalized in MCH were excluded from the study.

### Data Collection

Data collection and evaluation of Jiuzhaigou, Yushu, and Wenchuan earthquake patients were performed according to a standard protocol (10). Uniformed sheets were used to collect medical records of seismic inpatients both in MCH hospital and field hospitals from May 12th, 2008 to September 26th, 2017, and included general personal information, injury causes, injury location, injury type, injury severity, and method of treatment. General personal information and injury causes reported by patient self. For patients who are seriously injured and unable to communicate, we asked accompanying family members, rescuers, or witnesses. Microsoft EXCEL software was used for data input. Information collectors were trained prior to formal data collection.

Injury causes were based on the primary reason for the injury, such as hit/strike by objects, slips/falls, and building collapse/burying. The attending physician diagnosed injury location and injury type. Injury location and injury type were based on the final hospital diagnosis confirmed by the attending physician. Injury severity was evaluated using the Injury Severity Score (ISS), which is the most commonly used scoring system for assessing a patient's injury level during trauma care (11). The ISS is defined as the sum of the squares of the highest abbreviated injury scale-ISS in body regions with the highest ISS score (10). Classification of the severity of injuries was as follows: ISS <16, minor or moderate injury; ISS≥16, serious injury.

### Statistical Analysis

Categorical variables are presented as frequencies and percentages, and continuous variables are presented as averages, standard deviations, and median values. The Chi-square analysis was used for comparison of clinical variables between different parameters, and the Fisher exact test was used in cases where the patient number was <5). A *p* < 0.05 was considered statistically significant. Statistical analyses were performed using SPSS 17.0 software.

### Ethics

Our study was approved by the Ethical Committee of the Mianyang Central Hospital (Mianyang, China).

## Results

### Comparison of Clinical Parameters of Earthquake Patients From Three Large Earthquakes

As shown in Table 1, earthquake victims in Wenchuan (647 males and 545 females with a median age of 42.17), Yushu (85 males and 75 females, with a median age of 42.5), and Jiuzhaigou (25 males and 23 females with a median age of 36.7) are compared. Data showed that no significant differences were observed between gender (*p* > 0.05). However, a significant difference was observed in age (*p* < 0.05). We found that most of the patients in the three earthquakes were younger than 60 years old, especially in the Jiuzhaigou earthquake. In addition, educational backgrounds and occupations were also different in the three major earthquakes (*p* < 0.001, respectively). The majority wounded in the Wenchuan and Jiuzhaigou earthquakes have a high school education or above. In the Yushu earthquake, however, more patients are below the primary school. Medical staff and teachers were more vulnerable to the earthquake in Wenchuan and Jiuzhaigou earthquakes. But in the Yushu earthquake, most of the wounded were Farmers. Besides, building type and marital status were also different in the three earthquakes (*p* < 0.001, respectively). Most of the building types in the Wenchuan earthquake were concrete, whereas it was mud+concrete in the Jiuzhaigou and mud in the Yushu earthquake.

**Table 1:** The general characteristics of Wenchuan, Yushu, and Jiuzhaigou earthquake patients.

	**Wenchuan earthquake** ***N*** **=** **1182**		**Yushu earthquake** ***N*** **=** **160**		**Jiuzhaigou earthquake** ***N*** **=** **48**		
**Characteristics**	**Cases (*n*)**	**Percent (%)**	**Cases (*n*)**	**Percent (%)**	**Cases (*n*)**	**Percent (%)**	***p*-value^**1**^**
**Gender**							0.957
Male	637	53.9 %	85	53.1%	25	52.1%	
Female	545	46.1 %	75	46.9%	23	47.9%	
**Age(yr)**							0.023
≤60	932	78.8 %	136	85%	44	91.7 %	
>60	250	21.2 %	24	15%	4	8.3 %	
**Education**							<0.001
Above high school	418	35.4%	25	15.5%	35	34.4%	
Junior high school	562	47.5%	35	21.9%	10	43.7%	
Below primary school	202	17.1%	100	62.5%	3	21.9%	
**Occupation**							<0.001
Farmer	76	6.4%	79	49.4%	5	10.4%	
Teacher	313	26.5%	30	18.8%	0	0%	
Government officer	32	2.7%	2	1.3%	1	2.1%	
Worker	235	19.9%	27	16.9%	15	31.3%	
Medical staff	340	28.8%	10	6.3%	1	2.1%	
Student	60	5.1%	5	3.1%	20	41.7%	
Business man	78	6.6%	5	3.1%	5	10.4%	
Others	48	4.1%	2	1.3%	1	2.1%	
**Building type**							<0.001
Concrete	704	59.6%	24	15%	5	10.4%	
Mud+concrete	320	27.1%	41	25.6%	28	58.3%	
Mud	158	13.4%	95	59.4%	15	31.3%	
**Marital status**							<0.001
Single	469	39.7%	40	25%	37	77.1%	
Married	713	60.3%	120	75%	11	22.9	

### Comparison of the Injury Characteristics of Earthquake Patients From Three Large Earthquakes

Table 2 shows that the most common types of injury caused by these three earthquakes were different (*p* < 0.05). Wenchuan earthquake patients and Jiuzhaigou earthquake patients were primarily injured by the hit/strike of objects. However, most patients in the Yushu earthquake suffered from building collapse/burying-related injuries. Thus, the most common types of injury between the three earthquakes were different (*p* < 0.05). Most survivors of the Wenchuan and Jiuzhaigou earthquakes suffered from fractures, while the majority of survivors of the Yushu earthquake suffered from soft tissue injury. In addition, we showed that there was a significant difference in ISS as well as in treatment when comparing Jiuzhaigou earthquake patients to Wenchuan and Yushu earthquake patients (*p* < 0.05).

**Table 2:** The Injury characteristics of Wenchuan, Yushu, and Jiuzhaigou earthquake patients.

	**Wenchuan earthquake**		**Yushu earthquake**		**Jiuzhaigou earthquake**		
	**Cases (*n*)**	**Percent (%)**	**Cases (*n*)**	**Percent (%)**	**Cases (*n*)**	**Percent (%)**	***p*-value^**1**^**
**Injury causes**							<0.001
Hit/strike by objects	565	47.8 %	63	39.3%	24	50 %	
Slip/Falling	321	27.2 %	22	13.8%	10	20.8%	
Building collapse /Burying	187	15.8 %	67	41.9%	9	18.8%	
Others	109	9.2 %	8	5%	5	10.4%	
**Injury location**							0.059
Head /Face	202	17.1 %	12	7.5%	8	16.7%	
Chest/ spine	162	13.7 %	18	11.3%	7	14.5%	
Abdomen/Pelvis	102	8.6 %	15	9.4%	3	6.3%	
Arms / Legs	716	60.6 %	115	71.9%	30	62.5%	
**Injury types**							<0.001
Fractures	996	84.2 %	67	42%	34	71%	
Soft tissue injury	122	10.3 %	74	46.2%	12	25%	
Amputation	34	2.9%	9	5.6%	1	2%	
Others	30	2.6%	10	6.2%	1	2%	
**Injury severity**							<0.001
ISS <16	584	49.4%	128	80%	36	75%	
ISS ≥16	598	50.6%	32	20%	12	25%	
**Operation treatment**	910	77%	62	38.8%	28	58.3%	0.612
Orthopedic surgery	562	61.8%	43	69.4%	15	53.6%	
Neurosurgery	240	26.4%	13	21%	7	25%	
Plastic surgery	59	6.5%	3	4.8%	3	10.7%	
General surgery	49	5.4%	3	4.8%	3	10.7%	
**Non-operation**	272	23%	98	61.2%	20	41.7%	
In-hospital deaths	42		0		0		
length of hospital stays (Days)	12 ± 8.3		6.2 ± 4.3		6.7 ± 3.8		0.025

By comparing the treatment plans of this three earthquakes, we found that Wenchuan and Jiuzhaigou earthquake patients were mainly treated by surgery, while Yushu earthquake patients were mainly treated by non-operation treatment (*p* < 0.001). In overall surgical procedures, orthopedic surgery appeared to be the most frequent followed by neurosurgery, plastic surgery, and general surgery.

A total of 42 cases died in the Wenchuan earthquake and the mortality rate of hospitalized patients was 3.55%. The main causes of death were severe chest trauma and head injury (47%), crush syndrome (23%), infection (12%), and multiple organ failure (18%). length of hospital stay between these three earthquakes was different (*p* < 0.05), Wenchuan earthquake patients stay in the hospital longer.

### Comparison of the Surgical Treatment of Earthquake Patients From Three Large Earthquakes

In general, surgeries are needed for corresponding harm from earthquakes. According to our findings, open reduction and internal fixation (ORIF) took up the largest part of the operated orthopedic surgeries, and debridement was the second largest. Similarly, as the main neurosurgical surgery, decompressive craniectomy took on the highest frequency. In terms of plastic surgery, debridement accompanied with incision, split-skin graft, and infection drainage showed the top occurrence rate. Meanwhile, debridement and laparotomy occupied the most part the aspect of general surgery.

### The Time of the Different Injury Types and Surgical Treatment in Patients From Jiuzhaigou, Whenchuan and Yushu Earthquake

**Figure 3:**
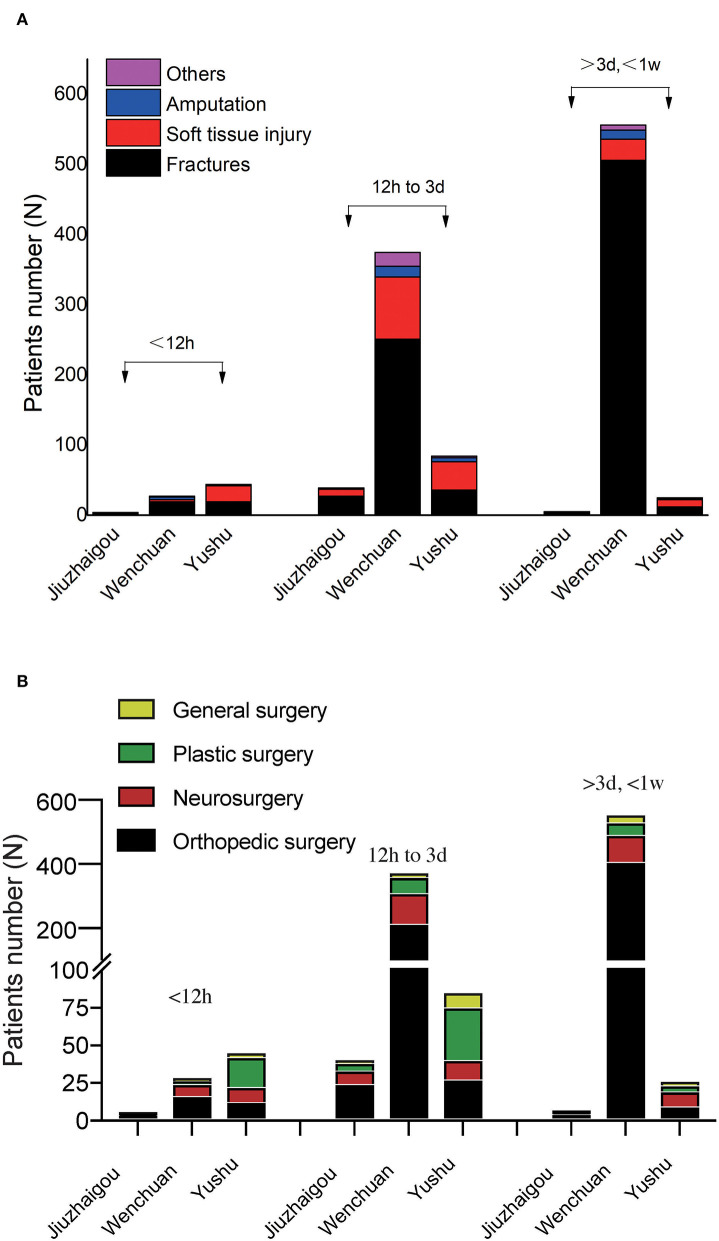
The time of the different injury types and surgical treatment in Jiuzhaigou, Whenchuan and Yushu earthquake patients. **(A)** The time of the different injury types in different earthquake patients. **(B)** The time of the different surgical treatment in different earthquake patients.

During the stages of final hospital diagnosis from 12 hours to a week, fractures were the predominant injury type of Jiuzhaigou and Wenchuan earthquake victims, whereas soft tissue injury was the main injury type among Yushu earthquake patients (Table 3; Figure 3A). Thus, a significant difference in injury type was observed among the patients of the three earthquakes. At higher levels of ground shaking earthquake, the evacuation of casualties is significantly delayed, which may be due to more serious traffic disruption. In contrast, in lower levels of ground shaking earthquakes and better traffic situations, patients may swarm into hospitals in a short time.

**Table 3:** Comparison of the surgical treatment of earthquake patients from three large earthquakes.

	**Wenchuan earthquake**		**Yushu earthquake**		**Jiuzhaigou earthquake**		
	**Cases (*n*)**	**Percent (%)**	**Cases (*n*)**	**Percent (%)**	**Cases (*n*)**	**Percent (%)**	***p*-value^**1**^**
**Orthopedic surgery**							0.014
Debridement	79	14.1%	12	27.9%	5	33.3%	
Amputation	135	24%	2	4.7%	0	0%	
External fixation	67	11.9%	6	14%	2	13.3%	
CR/casting/CRIF	84	14.9%	4	9.3%	1	6.7%	
ORIF open reduction/internal fixation	175	31.1%	18	41.9%	6	40%	
Other	22	3.9%	1	2.3%	1	6.7%	
**Neurosurgery**							0.853
Decompressive craniectomy	100	41.7%	7	53.8%	4	57.1%	
Cranioplasty	58	24.2%	2	15.4%	1	14.3%	
Craniotomy	72	30%	3	23.1%	2	28.6%	
Others	10	4.2%	1	7.7%	0	0%	
**Plastic surgery**							0.172
Debridement with split skin graft	34	57.6%	0	0%	1	33.3%	
Incision and drainage of infections	18	30.5%	2	66.7%	2	66.7%	
others	7	11.9%	1	33.3%	0	0%	
**General surgery**							0.182
Laparotomy	28	57.1%	0	0%	1	0%	
Debridement	15	30.6%	2	66.7%	2	100%	
Others	6	12.2%	1	33.3%	0	0%	

*CR, closed reduction; CRIF, closed reduction/internal fixation*.

We further analyzed the distribution of our hospital surgeries at different time points (Tables 4, 5; Figure 3B). Orthopedic surgery and neurosurgery were the most surgery methods for Jiuzhaigou and Wenchuan earthquake victims, but plastic surgery is relatively high in patients who survived Yushu earthquakes. Therefore, in the event of earthquakes, medical resources should be allocated to the Orthopedic surgery and neurosurgery department first.

**Table 4:** The time of the different injury types in Jiuzhaigou, Whenchuan and Yushu earthquake patients.

**Injury types**	**Occurrence time**					
	**<12h (*n*)**	***p***	**12h to 3d (*n*)**	***p***	**>3d, <1w (*n*)**	***p***
**Jiuzhaigou earthquake**		0.004		0.001		<0.001
Fractures	3		27		4	
Soft tissue injury	1		10		1	
Amputation	0		1		0	
Others	0		1		0	
**Wenchuan earthquake**						
Fractures	19		251		506	
Soft tissue injury	3		89		30	
Amputation	4		15		13	
Others	1		20		7	
**Yushu earthquake**						
Fractures	19		36		12	
Soft tissue injury	23		40		10	
Amputation	1		6		2	
Others	1		2		1	

**Table 5:** The time of the different surgical treatment in Jiuzhaigou, Whenchuan and Yushu earthquake patients.

**Surgery**	**Occurrence time**					
	**<12h (*n*)**	***p* ^1^**	**12h to 3d (*n*)**	**P^**1**^**	**>3d, <1w (*n*)**	***p* ^1^**
**Jiuzhaigou earthquake**		0.002		<0.001		<0.001
Orthopedic surgery	2		23		3	
Neuro surgery	1		9		1	
Plastic surgery	0		5		1	
General surgery	1		2		0	
**Wenchuan earthquake**						
Orthopedic surgery	15		214		407	
Neuro surgery	8		96		85	
Plastic surgery	2		50		39	
General surgery	2		15		25	
**Yushu earthquake**						
Orthopedic surgery	11		26		8	
Neuro surgery	10		13		10	
Plastic surgery	20		35		4	
General surgery	3		10		3	

## Discussion

Earthquakes have become a major cause of death and disability worldwide (12). In the past decade, a series of earthquakes occurred in Western China and it is believed that the next big earthquake is around the corner (2, 13, 14). These large-scale catastrophes are a major challenge for medical systems. Therefore, medical organizations need to summarize their experience regarding medical rescue after an earthquake, to provide information for similar future catastrophes. In China, the Chinese medical system has a three-tiered system(15, 16). The bottom of this system is the rural sector. There are village stations, township health centers, and county hospitals in this area, which have only a few well-trained medical staff with little medical equipment or medications. They provide preventive and primary care services. In the middle of this system is the urban sector, including street health stations, community health centers, and district hospitals. These medical places have about thirty beds with some qualified medical staff, who generally handle common illnesses and general emergencies. The most serious illnesses are referred to the third and final tier, the city or regional hospitals which have the best medical staff and are equipped with modern facilities. In the event of a serious accident or natural disaster, patients will be referred directly to the city or regional hospitals. Furthermore, these hospitals also will organize professional medical teams to the accident site. China has free public healthcare based on social insurance plans (17). Government subsidies (about 70% of the total funds) covered rural and urban resident-based health insurance and employer and employee contributions covered employee-based health insurance. Casualties caused by natural disasters, such as earthquakes, all medical costs are covered by the government, including all Chinese and foreigners.

In this study, MCH, one of the best city hospitals in China's Sichuan province, has provided medical support to over 1,500 earthquake-related patients from three major earthquakes (Jiuzhaigou, Yushu, and Wenchuan earthquakes). Therefore, we believe that MCH injury analysis is highly representative and reliable.

### Special Occupations Are at High Risk Among Earthquake Victims

The clinical parameters of patients who survived Wenchuan, Yushu, and Jiuzhaigou earthquake showed that compared to patients who survived the Jiuzhaigou earthquake they were relatively young (36.7 year old), and their age difference was significantly from the age of patients of Wenchuan (42.17 year old) and Yushu earthquake (42.5 year old). This may be because most Jiuzhaigou earthquake patients were tourists, whereas, in the Yushu and Wenchuan earthquakes, most patients were residents. This reason can also explain the difference in marital status between these the patients who survived earthquake. Interestingly, there was a higher possibility that medical and educational workers get harmed in Yushu and Wenchuan earthquakes. The reason lies in that they tended to save others' lives than their own. Teachers bear more responsibility for the safety of students, while doctors and nurses show up and offer help in seriously hit regions. These all render them at a higher risk of getting injured. But the most injured in the Jiuzhaigou earthquake are students, this is because firstly Jiuzhaigou is a tourist attraction, and August is the summer vacation, so many students travel there.

### Building Characteristics Relevant to the Risk of Injury and Four Limbs Were More Frequently Involved in Fracture

Through analysis of injury characteristics of earthquake patients, injuries among the Jiuzhaigou earthquake patients were mostly due to hit/strike of objects (50%), and this kind of injury causes also had a high proportion (47.8%) in Wenchuan earthquake patients. Our result was in accordance with the findings of a previous report (18). Furthermore, we determined that 20.8% of injuries were caused by slips/falls. This was not only observed in the Jiuzhaigou earthquake but also in others (18), and may be due to the fact that individuals jumped from buildings or chose other dangerous escape routes, which caused unnecessary injuries during the earthquake and the aftershock. This was also found in patients who survived the Wenchuan earthquake (27.2%) (27.), and strongly shows that detailed instruction methods and self-rescue skills during natural disasters are essential. In patients who survived the Yushu earthquake, most injuries were related to building collapse/burying (41.9%), which was statistically significant when compared to Jiuzhaigou and Wenchuan earthquakes (*p* < 0.05). This may be because Yushu is a poverty-stricken area in China where people live under poor conditions. Most buildings were prepared from mud (59.4%), which could easily collapse during an earthquake. Therefore, building collapse or burying was the predominant cause of injuries in patients from Yushu. Most buildings in Wenchuan and Jiuzhaigou were tall buildings from reinforced concrete (59.6 and 10.4%, respectively) or mud + concrete (27.1 and 58.3%, respectively) during an earthquake, something could fall from the building, thereby, injuring individuals by hit/strike of objects. In addition, Wenchuan and Jiuzhaigou are considered mountain areas, and building collapse and falling rocks are common geohazards in these mountainous areas. Lastly, Wenchuan and Jiuzhaigou earthquakes occurred at night and in the early morning. People's field of vision was limited and it was impossible to accurately judge the falling objects. Thus, individuals in Wenchuan and Jiuzhaigou earthquakes are more likely to be injured by the hit/strike of objects, especially during an earthquake.

In this study, we showed that arms and legs were the most frequently injured locations, which in Jiuzhaigou, Yushu, and Wenchuan earthquake patients accounted for an injury percentage of 62.5, 72, and 60.6%, respectively. These findings were consistent with data presented in a previous report (19–21). Head, chest, and spine also reached a certain injury percentage (all above 10% in three earthquakes), indicating that such types of injuries should not be neglected when more attention is being paid to the management of limb injuries. In several studies, it has been demonstrated that the body posture and living status of patients at the time of a disaster could have an impact on the incidence of fractures and injuries during an earthquake (22).

### Severe Soft Tissue Injuries Cannot Be Ignored

Additionally, our data showed that fractures accounted for 42% of injuries, whereas soft tissue injuries such as blunt trauma and bruise counted for 46.2% of injuries in patients who survived the Yushu earthquake. These findings were not consistent with Wenchuan and Jiuzhaigou earthquakes, in which patients with fractures reached over 70%. The possible reason is most Yushu earthquake patients were caused by the collapse of mud or wooden buildings and burying, resulting in more soft tissue injury. Although soft tissue injuries are not fatal, they can also cause serious infections, myoglobinemia, malnutrition, and internal environmental disorders. The mortality rate of patients with severe infections and crush syndrome will also increase significantly. However, in most Wenchuan and Jiuzhaigou earthquake patients were caused by hit/strike by heavy objects (47.8 and 50%, respectively), resulting in fractures. It is important to note that fracture injuries may occur both during and after an earthquake. These results may help emergency response centers predict the type of patient that needs treatment and thereby lead to a more rational allocation of limited medical resources. In addition, based on the ISS, patients were classified as suffering from mild or moderate and severe injuries. In our study, 75% patients of Jiuzhaigou patients had an ISS score <16, and only 25% of patients had an ISS score ≥16, which was similar to that of Yushu earthquake patients. However, in patients of the Wunchuan earthquake, the ISS score ≥16 reached 50.6%. The difference of injury severity between patients who survived the Wenchuan, Yushu, and Jiuzhaigou earthquakes was extremely significant (*p* < 0.001). This may be because both Yushu and Jiuzhaigou earthquakes were less powerful compared to the Wenchuan earthquake.

Through the comparison of the traumatic conditions of the injured in the three earthquakes, we found that different levels of ground shaking during earthquakes might lead to the difference in the injury mechanism of the injured from the aspect of earthquake medical rescue. The higher level of ground shaking is more severe the damage to the houses and the more obvious the rockfall in the mountains. Consequently, the proportion of those injured by heavy objects will increase significantly in earthquakes with higher levels of ground shaking. In the meantime, it is also necessary to consider the geographical location of the earthquake, economic conditions, and housing structure, which can also lead to different injury mechanisms.

### Surgical Treatment Is Still the Main Type of Treatment

According to the epidemiology about traumas caused by earthquakes put forward by Ramirez et al. (23), the quantities and kinds of harms resulting from earthquakes can be different based on mankind's reasons, context, and geographic factors. Nevertheless, the injuries from earthquakes are actually in bones mostly, especially the fractures of long bones are more common. Our data also showed that surgical treatment was the main type of treatment for Jiuzhaigou (58.3%) and Wenchuan earthquake patients (77%). Out of various surgical cases, the majority were related to orthopedics around 60% and the remaining 40% were non-orthopedics in nature (neurosurgery about 20%, plastic surgery about 10%, general surgery about 10%). In contrast, only a small number of Yushu earthquake patients (38.8%) underwent surgery, although many patients ended up with fractures or other serious injuries. This difference was statistically significant (*p* < 0.05). Most individuals in Yushu are Tibetans, who are important members of the Chinese nation. This population has its own unique culture and medicine named Tibetan Medicine (24). Many patients who survived the Tibetan earthquake chose and trusted Tibetan Medicine over surgical treatment. Although surgery was the treatment of choice in most patients with fractures, a small percentage of patients who survived earthquake did receive non-surgical treatment. These results indicated that surgical teams are essential during the immediate aftermath of an earthquake, and that general medical teams are also essential.

### Internal Fixation, Decompressive Craniectomy, and Debridement With Split Skin Grafting Were Common Surgery

In accordance with a deeper study on surgical therapies, the rate of open reduction and internal fixation (ORIF) ranks top in the area of orthopedic surgery (31– 42% in three earthquakes), and debridement the second (14–33% in three earthquakes). Based on prior research, organ failure and pulmonary complications could be greatly reduced by giving early fixation, which would contribute to a higher rate of survival. However, early definitive treatment for multiple injury patients may have side effects, indicating that the best time of operation for multiple injury patients is 2–5 days after injury. In Neurosurgery, decompressive craniectomy (42–57% in three earthquakes) was the commonest surgery performed in our study. Here it appears to be the second most common surgery after orthopedics in the post-earthquake scenario. Nonetheless, patients with multiple injuries may suffer from side effects if they are given definitive treatment early. This means that 2 to 5 days after trauma should be the optimum operating time for them. On the basis of our research, decompressive craniectomy is the most commonly operated in the field of neurosurgery. However, it falls to the second performed after orthopedics if it is a post-earthquake situation. In a similar study of the 1999 Taiwan earthquake (25), around 30% of people died from head injuries. Among all plastic surgical cases in our study, debridement with split skin grafting accounted majority (33–58% in Jiuzhaigou and Wenchuan earthquakes). Similarly in a study by Shrestha et al. (26), 22% of the total operations were for soft tissue injury and the most common surgery was split skin grafting in general surgery, laparotomy, and debridement accounted for 30–100% in our study. Similar findings in studies by Zhang (27) and Wolf (28).

In our study, only Wenchuan documented 42 cases of mortality (0.4%). The main causes of death were severe head injury, crush syndrome, infection, and multiple organ failure. Especially, chest trauma and craniocerebral trauma tend to cause patients to die quickly within a short period of time after the injury. Therefore, the post-earthquake treatment of patients with chest or craniocerebral trauma greatly affects their therapeutic effects.

### Burst Increase in Admission

Analyze the length of hospital stay, treatments are mainly given to the patients directly transferred from the epicenter in the early stage after the earthquake, and the severe patients from other front-line hospitals in the later stage. During a considerable period of time, patients may arrive at hospitals successively. Nonetheless, there may not be an obvious peak in the speed of treatment, which requires hospitals to rationally arrange the allocation of medical resources to avoid the bad consequences caused by the underestimation of the arrival of the wounded in the later stage of rescue. For earthquakes with lower magnitude and better traffic situations, patients may pour in in a short time. As they can all move outward in a short period of time, it is hard for the continued flow of the wounded to happen. In our study, we found out that a low ground shaking earthquake or earthquake area has better traffic conditions than Jiuzhaigou and Yushu earthquakes, most patients (about 80%) arrived at the hospital in a short period of time (<3 days). For this reason, hospitals should make enough preparation in advance to avoid the deficient treatment resulting from insufficient medical resources in the early stage of rescue.

It is worth noting that injuries may be further aggravated by traffic, climate problems, and geological. Infections frequently happen among the injured victims during transportation. Yang et al. (29) indicated that the infections were intrinsically related to the longer rescue time and delayed transportation and open injury had a higher infection possibility by transportation. We also notice that infections in transit will affect the outcome of multiple bone-fracture crush injuries or amputation and crush patients. In addition, some neurologic injuries, multiple bone-fracture crush injuries, and crush syndrome were worsened by improper transportation. Therefore, we cannot ignore the medical protection during the transfer of patients.

### Limitations

Although the results of this study provide important information for the clinical management of earthquake-injured patients, there are some limitations. First, in this retrospective study, the medical records of earthquake patients were analyzed, which were created by many different doctors. It is impossible to verify the intra-doctor and inter-doctor variability in making a diagnosis. Hospital medical record keeping was another issue in serious disaster situations. Detailed medical records of all hospitalized patients were often incomplete because of the initial disorganization, especially during the first few hours after an earthquake. In addition, some patients had been treated in other hospitals, which may lead to a selection bias in the data. Lastly, since our research is based on the patients only in our hospital, the research has certain limitations. However, MCH hospital was the major provider of medical services to patients in the Wenchuan, Lushan, and Jiuzhaigou earthquakes, and over 1,000 earthquake victims were admitted to our hospital. Therefore, although a number of records are missing, we are confident about the accuracy and authority of our data.

## Conclusions

In our study, we found that different geographic locations, populations, and religions need a tailored approach to the rescue effort. First, the geophysical damage degree exerts an obvious influence on the injury mechanism of earthquake casualties. In earthquakes of high magnitude, the number of those injured by heavy objects and total burying rises greatly. Correspondingly, there is a reduction in fall injuries for patients. However, it results in more serious illnesses and an even higher death rate. Besides, the geophysical damage degree clearly affects the evacuation of the injured after the earthquake. The higher the earthquake magnitude is, the later the fastigium of patient evacuation to the hospital would be, and there may even be no obvious peak. Nevertheless, evacuation time can be significantly prolonged, and there will be a lasting impact on the work of hospitals. Correspondingly, in earthquakes of lower magnitude, the impact of which on casualty evacuation is small, the fastigium of their arrival in hospital is obviously earlier, the duration of patient flow shorter, and the time of influence on hospital work less. In the field of earthquake medical rescue, the strength of orthopedics is absolutely the most important, but it is also necessary to strengthen the rescue forces of thoracic neurosurgery and general surgery. Lastly, the popularization of correct knowledge of emergency shelters will help to reduce the damage caused by blindly jumping or escaping while an earthquake happens. We hope that the results of this study will be of use in future natural disasters in China and the rest of the world.

## Data Availability Statement

The raw data supporting the conclusions of this article will be made available by the authors, without undue reservation.

## Ethics Statement

The studies involving human participants were reviewed and approved by Ethical Committee of the Mianyang Central Hospital. Written informed consent to participate in this study was provided by the participants' legal guardian/next of kin.

## Author Contributions

DW and XD designed experiments. SX, BS, and JY carried out experiments and took the photos. SX, BS, MH, and PY analyzed experimental results. SX, WX, and GL wrote the manuscript. SX and ZS revised the manuscript. All authors contributed to the article and approved the submitted version.

## Conflict of Interest

The authors declare that the research was conducted in the absence of any commercial or financial relationships that could be construed as a potential conflict of interest.

## Publisher's Note

All claims expressed in this article are solely those of the authors and do not necessarily represent those of their affiliated organizations, or those of the publisher, the editors and the reviewers. Any product that may be evaluated in this article, or claim that may be made by its manufacturer, is not guaranteed or endorsed by the publisher.

## References

[1] Li J Böse M Wyss M Wald DJ Hutchison A Clinton JF. Estimating rupture dimensions of three major earthquakes in Sichuan, China, for early warning and rapid loss estimates. Bullet Seismol Soc Am. (2020) 110:920–36. 10.1785/0120190117

[2] Yin YP Wang F Ping S. Landslide hazards triggered by the 2008 Wenchuan earthquake, Sichuan, China. Landslides. (2009) 6:139–52. 10.1007/s10346-009-0148-5

[3] Kang P Zhang L Liang W Zhu Z Liu Y Liu X. Medical evacuation management and clinical characteristics of 3,255 inpatients after the 2010 Yushu earthquake in China. J Trauma Acute Care Surg. (2012) 72:1626–33. 10.1097/TA.0b013e3182479e07 22695432

[4] Tang J Yao Y Zhang L. Temporal and spatial ionospheric variations of 20 April 2013 earthquake in Yaan, China. IEEE Geosci Remote Sens Lett. (2015) 12:2242–6. 10.1109/LGRS.2015.2463081

[5] Gaulke LS Xiao W Scanlon A Henck A Hinckley T. Evaluation criteria for implementation of a sustainable sanitation and wastewater treatment system at Jiuzhaigou national park, Sichuan Province, China. Environ Manage. (2010) 45:93–104. 10.1007/s00267-009-9398-1 19924471

[6] Qiao X Xiao W Jaffe D Kota SH Ying Q Tang Y. Atmospheric wet deposition of sulfur and nitrogen in Jiuzhaigou national nature reserve, Sichuan Province, China. Sci Total Environ. (2015) 511:28–36. 10.1016/j.scitotenv.2014.12.028 25525712

[7] Noji E.K.. The public health consequences of disasters. Prehosp Disaster Med. (2000) 15:21–31. 10.1017/S1049023X00025255 11227602

[8] Bulut M Fedakar R Akkose S Akgoz S Ozguc H Tokyay R. Medical experience of a university hospital in Turkey after the 1999 Marmara earthquake. Emerg Med J. (2005) 22:494–8. 10.1136/emj.2004.016295 15983085 PMC1726859

[9] Peek-Asa C Kraus JF Bourque LB Vimalachandra D Yu J Abrams J. Fatal and hospitalized injuries resulting from the 1994 Northridge earthquake. Int J Epidemiol. (1998) 27:459–65. 10.1093/ije/27.3.459 9698136

[10] Papadopoulos IN Kanakaris N Triantafillidis A Stefanakos J Kainourgios A Leukidis C. Autopsy findings from 111 deaths in the 1999 Athens earthquake as a basis for auditing the emergency response †. Br J Surg. (2004) 91:1633–40. 10.1002/bjs.4752 15505869

[11] Kang P Tang B Liu Y Liu X Shen Y Liu Z. Profile and procedures for fractures among 1,323 fracture patients from the 2010 Yushu earthquake, China. Am J Emerg Med. (2016) 34:2132–9. 10.1016/j.ajem.2016.07.064 27543441

[12] Ellidokuz H Ucku R Aydin UY Ellidokuz E. Risk factors for death and injuries in earthquake: cross-sectional study from Afyon, Turkey. Croat Med J. (2005) 46:613. 16100765

[13] Qu Y Huang C Zhang P Zhang J. Microblogging after a major disaster in China: a case study of the 2010 Yushu earthquake, ACM Conference on Computer Supported Cooperative Work, CSCW 2011, Hangzhou, China, March, 2011, pp. 25-34.

[14] Li C Luo X Zhang W Zhou L Wang H Zeng C. YaAn earthquake increases blood pressure among hospitalized patients. Clin Exp Hyperten. (2016) 38:495. 10.3109/10641963.2015.1116549 27398731

[15] Yi B. An overview of the Chinese healthcare system. Hepatobiliary Surg Nutr. (2021) 10:93. 10.21037/hbsn-2021-3 33575292 PMC7867737

[16] Hsiao WC. The Chinese health care system: lessons for other nations. Soc Sci Med. (1995) 41:1047–55. 10.1016/0277-9536(94)00421-O 8578327

[17] Meng Q Mills A Wang L Han Q. What can we learn from China's health system reform? Bmj. (2019) 365:12349. 10.1136/bmj.l2349 31217222 PMC6598719

[18] Qiu J Liu GD Wang SX Zhang XZ Zhang L Li Y. Analysis of injuries and treatment of 3,401 inpatients in 2008 Wenchuan earthquake–based on Chinese trauma databank. Chin J Traumatol. (2010) 13:297–303. 20880457

[19] Bai XD Liu XH. Retrospective analysis: the earthquake-injured patients in Barakott of Pakistan. Chin J Traumatol. (2009) 12:122–4. 19321059

[20] Zhang L Li H Carlton JR Ursano R. The injury profile after the 2008 earthquakes in China. Injury-Int J Care Injur. (2009) 40:84–6. 10.1016/j.injury.2008.08.045 19117564

[21] Salimi J Abbasi M Khaji A Zargar M. Analysis of 274 patients with extremity injuries caused by the Bam earthquake. Chin J Traumatol. (2009) 12:10–3. 19159509

[22] Kang P Tang B Liu Y Liu X Liu Z Lv Y. Medical efforts and injury patterns of military hospital patients following the 2013 Lushan Earthquake in China: a retrospective study. Int J Environ Res Public Health. (2015) 12:10723–38. 10.3390/ijerph120910723 26334286 PMC4586639

[23] Ramirez M Peek-Asa C. Epidemiology of traumatic injuries from earthquakes. Epidemiol Rev. (2005) 27:47–55. 10.1093/epirev/mxi005 15958426

[24] Loizzo JJ Blackhall LJ Rapgay L. Tibetan medicine. Ann N Y Acad Sci. (2009) 1172:218–30. 10.1196/annals.1393.008 19743556

[25] Shin TC Teng TL. An overview of the 1999 Chi-Chi, Taiwan, earthquake. Bull Seismol Soc Am. (2001) 91:895–913. 10.1785/012000073816892885

[26] JM S. Role of Plastic Reconstructive Surgery in the management of earthquake victims of Nepal in Tribhuvan University Teaching Hospital. J Inst Med. (216) 38:88.

[27] Zhang J Ding W Chen A Jiang H. The prominent role of plastic surgery in the Wenchuan earthquake disaster. J Trauma Acute Care Surg. (2010) 69:964–9. 10.1097/TA.0b013e3181e9f0e0 20938280

[28] Wolf Y Bar-Dayan Y Mankuta D Finestone A Onn E Morgenstern D. An earthquake disaster in Turkey: assessment of the need for plastic surgery services in a crisis intervention field hospital. Plastic Reconst Surg. (2001) 107:163–8. 10.1097/00006534-200101000-00026 11176618

[29] Yang C Wang HY Zhong HJ Zhou L Jiang DM Du DY. The epidemiological analyses of trauma patients in Chongqing teaching hospitals following the Wenchuan earthquake. Injury. (2009) 40:4889–2. 10.1016/j.injury.2009.01.102 19328487

